# Candidemia caused by *Candida haemulonii*: a case report and literature review in neonates

**DOI:** 10.1590/S1678-9946202567042

**Published:** 2025-07-07

**Authors:** Minxue Liu, Chunyun Fu, Xingchun Chen

**Affiliations:** 1 Maternal and Child Health Hospital of Guangxi Zhuang Autonomous Region, Children’s Hospital, Department of Laboratory Medicine, Nanning, Guangxi, People’s Republic of China; 2 Guangxi Academy of Medical Sciences, The People’s Hospital of Guangxi Zhuang Autonomous Region, Department of Laboratory Medicine, Nanning, Guangxi, People’s Republic of China

**Keywords:** Candida haemulonii, Neonates, Candidemia, Emerging yeast pathogen, Antifungal susceptibility testing

## Abstract

Candidemia poses a significant challenge for hospitalized neonates with an increase in morbidity and mortality. However, candidemia caused by *Candida haemulonii* in newborns is rare but fatal. We report such a case in China and performed a literature review. A neonate with a gestational age of 31+6 weeks and a birth weight of 1,420g was diagnosed with *C. haemulonii* candidemia. The infectious agent was identified by matrix-assisted laser desorption/ionization time-of-flight mass spectrometry and sequencing of the internal transcribed spacer region. The *in vitro* antifungal susceptibility testing indicated high minimal inhibitory concentrations for fluconazole (>128 µg/mL), voriconazole (>8 μg/mL), and amphotericin B (>4 µg/mL). Fortunately, the newborn was successfully treated with fluconazole. After a literature review of *C. haemulonii* candidemia, we found that the risk factors of the candidemia might involve premature, low birth weight, invasive therapeutic devices, broad-spectrum antimicrobial agents, and parenteral nutrition infusion. This study will broaden our knowledge on neonatal candidemia caused by *C. haemulonii*.

## INTRODUCTION

*Candida haemulonii* has emerged as a significant fungal pathogen that can cause life-threatening invasive candidiasis worldwide^1^. Unlike *Candida auris*, another concerning emerging pathogen, *C. haemulonii* maintains a relatively low prevalence (0.9–1.7%)^2^. Since the first documented neonatal outbreak in 2007^3^, cases of *C. haemulonii* candidemia in neonates have been increasingly reported worldwide. Notably, *C. haemulonii* species from neonatal blood culture resistant to amphotericin B and fluconazole have been described^3,4^. We describe a case of *C. haemulonii*-associated neonatal candidemia in Guangxi, China. While the isolate showed *in vitro* resistance to fluconazole, clinical treatment outcomes were favorable. Additionally, we reviewed the cases of neonatal *C. haemulonii* candidemia in the literature.

### Ethics

This study was approved by the local Ethics Committee of The Maternal and Child Health Hospital of Guangxi Zhuang Autonomous Region. As the participants were children, their guardians gave informed consent on the participant’ behalf before the start of this study.

## CASE REPORT

A male neonate was delivered by Cesarean section at 31+6 weeks gestation due to intrauterine hypoxia. He had a birth weight of 1,420 g and an Apgar score of 7. He was immediately admitted to a county-level hospital neonatal intensive care unit (NICU). Then, hyperalimentation and tracheal intubation ventilation were started for treating abdominal distention and respiratory distress syndrome. Antibiotic prophylaxis included intravenous penicillin sodium (6 mg/kg/day) for 4 days and intravenous piperacillin-tazobactam (240 mg/kg/day) for 20 days after admission. Subsequently, prophylactic intravenous imipenem (80 mg/kg/day) was given for another 10 days. Despite these interventions, the newborn showed no clinical improvement and developed severe thrombocytopenia (platelet count 23,000/mm^3^). He was subsequently transferred to the NICU at the Guangxi Zhuang Autonomous Region Maternal and Child Health Hospital for advanced care.

Subsequent laboratory investigations showed elevated inflammatory markers, including 38.78 mg/L of C-reactive protein (CRP), of 0.43 ng/mL procalcitonin (PCT), and > 600 pg/mL of (1→3)-β-D-glucan. Given these findings, a blood culture was obtained to investigate suspected fungal infection.

**Figure 1:**
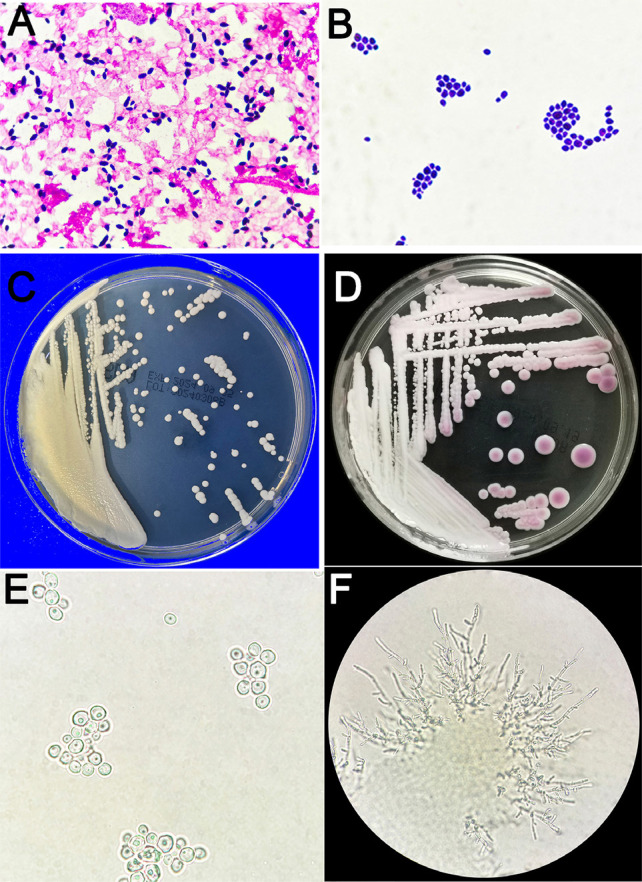
(A and B) Micromorphology examination showed ovoid to elongated, budding yeast-like spores; (C) The colonies on Sabouraud glucose agar after four days incubated at 35 °C; (D) The colonies on CHROMagar appeared as pink; (E) No germ tube was observed in serum after three h incubated at 37 °C; (F) Pseudohyphae.

After 52 h of incubation, the aerobic blood culture yielded positive results. Microscopic examination showed ovoid to elongated budding yeast-like positive spores (Figure 1A). The clinical isolate was sub-cultured on CHROMagar Candida plates in Sabouraud glucose agar and incubated at 35 and 42 °C, respectively. After four days incubating at 35 °C, it grew into smooth from white-to-cream colonies on Sabouraud glucose agar (Figure 1C) and pink on the CHROMagar Candida medium (Figure 1D). Microscopic examination confirmed similar morphological characteristics (Figure 1B). No growth was observed at 42 °C. The serum germ tube test of the species was negative (Figure 1E). Pseudohyphae could be observed under microscopy if inoculated on Mueller-Hinton agar and incubated at 35 °C for 48 h (Figure 1F).

The strain was initially identified as *C. haemulonii* (51% ) and *C. duobushaemulonii* (49% ) by the automated VITEK-2 compact system (version 9.02, bioMérieux). However, it was confirmed as *C. haemulonii* by matrix-assisted laser desorption/ionization time-of-flight mass spectrometry (MALDI-TOF MS; VITEK MS, bioMérieux) and internal transcribed spacer (ITS) sequencing, with 100% nucleotide identity to reference sequences in the NCBI GenBank database. The ITS sequence of this *C. haemulonii* isolate has been deposited into GenBank (accession Nº pp972761).

Antifungal susceptibility testing (AFST) was performed by using the DL-96 Fungus (Zhuhai DL Biotech, China) and the ATB Fungus-3 (bioMérieux), following the broth microdilution method based on Clinical and Laboratory Standards Institute M27^5^ to determine the Minimum inhibitory concentrations (MICs) of fluconazole, itraconazole, voriconazole, amphotericin B, caspofungin, micafungin, and 5-flucytosine in which *Candida parapsilosis* (ATCC 22019) served as the quality control strain. Results showed the high MICs of the isolate toward fluconazole, itraconazole, voriconazole, and amphotericin B and low MICs to 5-flucytosine, caspofungin, and micafungin (Table 1). To date, in accordance with Clinical and Laboratory Standards Institute M57S, epidemiological cutoff values have been established for *C. haemulonii* only for fluconazole, voriconazole, and anidulafungin, with no current clinical breakpoints^6^.

**Table 1:** Minimum inhibitory concentrations (MICs) of antifungal drugs.

	5-FC	FLZ	ITC	VRC	CAS	MFG	AMB
MIC (μg/mL)	0.25 (NE)	>128 (NWT)	1 (NE)	8 (NWT)	0.25 (NE)	0.12 (NE)	>4 (NE)

5-FC = 5-flucytosine; FLZ = fluconazole; ITC = itraconazole; VRC = voriconazole; CAS = caspofungin; MFG = micafungin; AMB = amphotericin B; NWT = non-wild-type; NE = breakpoint or epidemiological cutoff value not established.

**Figure 2:**
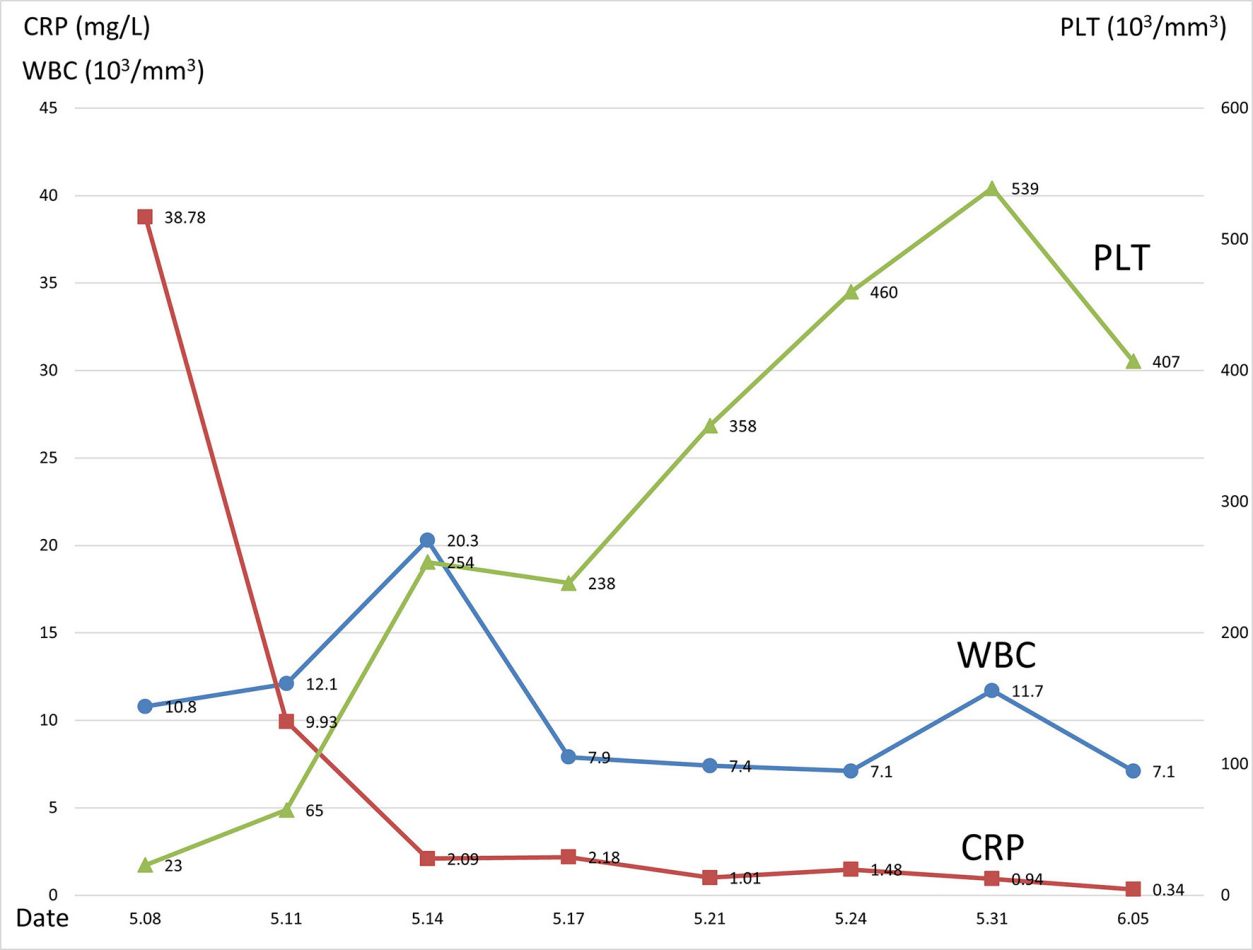
The change of PLT, WBC, and CRP during hospitalization. Intravenous fluconazole was given from May 9 to June 5. PLT = platelet; WBC = white blood cell; CRP = C-reactive protein.

Prior to confirming candidemia diagnosis, the neonate received empirical intravenous fluconazole therapy (12 mg/kg/day) due to his elevated (1-3)-β-D-glucan level (> 600 pg/mL). Following a five-day treatment course, the patient recovered, showing a platelet count 254,000/mm^3^, a CRP level of 2.09mg/L, and negative blood cultures. Despite the AFST showed resistance to fluconazole, the patient received intravenous fluconazole (12 mg/kg/day) for three weeks due to its favorable clinical response until his platelet count and other inflammatory markers turned normal, after which the patient was discharged (Figure 2).

## DISCUSSION

The *C. haemulonii* complex has been described including four opportunistic human pathogens: *C. haemulonii sensu stricto*, *C. pseudohaemulonii*, *C. vulturna*, and *C. duobushaemulonii* ^7,8^. *C. auris* is its phylogenetically related species^9^. It is difficult to differentiate *C. haemulonii* from *Candida auris* based on their morphology*.* However, the pseudohyphae of *C. haemulonii* may occur on conventional medium such as Mueller-Hinton agar, whereas *C. auris* typically does not^10^. Additionally, *C. haemulonii* fails to grow at 40 °C, whereas *C. auris* has a maximum growth temperature of 42 °C, which has become a useful differential characteristic^11^. Traditional biochemical methods often fail to accurately differentiate *C. haemulonii* from its closely related species. Reliable identification requires advanced techniques such as MALDI-TOF MS or molecular diagnostics^11-13^.

We found 19 neonatal cases of *C. haemulonii* candidemia in the literature^3,4,14-17^ (Table 2), all (100%) of which occurred in preterm infants, with 89.5% (17/19) having low birth weight. Most cases (94.7%, 18/19) had exposure to broad-spectrum antibiotics and invasive devices (including peripherally inserted central, central venous, or umbilical venous catheters). Parenteral nutrition was administered in 94.4% (17/18) of cases, and 82.4% (14/17) required mechanical ventilation. Our literature review showed a mortality rate of 15.8% (3/19) in pediatric patients with *C. haemulonii* candidemia, whereas 84.2% (16/19) of patients showed clinical improvement following treatment.

**Table 2:** Cases of candidemia in neonate caused by *Candida haemulonii* reported in the literature.

Article	Region	Gestation /Gender	Birth weight (g)	Prior antimicrobial agent exposure	Parenteral nutrition	Mechanical ventilation	Venous catheter	Delivery mode	Underlying condition	Treatment	Outcome
Khan *et al.* ^3^	Kuwait City, Kuwait	35w/M	2570	AMK, AMP, Tazobactam	Y	Y	CVC	NS	Prematurity, congenital diaphragmatic heria	Liposomal AMB/CAS/ FLZ	Death
		26w/M	710	AMK, AMP, VA	Y	Y	CVC	NS	Prematurity	FLZ	Clinical improvement
		25w/F	800	AMK, AMP, VA, CLO, TZP	Y	Y	CVC	NS	Prematurity	AMB / CAS	Clinical improvement
		31w/F	1035	AMK, AMP, MEM	Y	Y	CVC	NS	Prematurity	AMB	Death
Silva *et al.* ^4^	Pernambuco, Brazil	28w/M	1020	Yes，NS	NS	NS	NS	NS	Prematurity	AMB / FLZ	Clinical improvement
Jie *et al.* ^14^	Wenzhou, China	30+2 w/M	1500	MEM	Y	Y	PICC	Cesarean section	Prematurity	FLZ /VRC	Clinical improvement
		27+5 w/F	945	MEM, CPS	Y	Y	PICC	Vaginal delivery	Prematurity	FLZ	Death
		25+3 w/F	845	MEM, CPS	Y	Y	PICC	Vaginal delivery	Prematurity	FLZ	Clinical improvement
		34 w/M	1500	CPS	Y	Y	PICC	Cesarean section	Prematurity, congenital gastric wall defects	FLZ	Clinical improvement
		34 w/M	1000	MEM, CPS	Y	Y	PICC	Cesarean section	Prematurity, congenital heart disease	FLZ /VRC	Clinical improvement
		26+4 w/F	890	CPS	Y	Y	PICC	Cesarean section	Prematurity, tracheomalacia	FLZ	Clinical improvement
		30+1 w/M	970	CPS	Y	Y	PICC	Vaginal delivery	Prematurity, Congenital heart disease	FLZ /VRC	Clinical improvement
		37+1 w/M	2055	TZP	Y	N	PICC	Vaginal delivery	Congenital anal atresia	FLZ /VRC	Clinical improvement
Zhong *et al.* ^15^	Xiamen, China	25+1w/F	725	CPS, MEM, VA	Y	Y	PICC	Vaginal delivery	Prematurity	FLZ /MFG	Clinical improvement
		30w/M	1000	CPS, MEM	Y	N	PICC	Cesarean section	Prematurity	FLZ / MFG	Clinical improvement
		30w/F	1070	CPS, TZP	Y	N	PICC	Cesarean section	Prematurity	FLZ / MFG	Clinical improvement
Silva *et al.* ^16^	Recife, Brazil	26+4w/F	660	PEN, GM, TZP, AMK	Y	NS	Umbilical venous catheter	NS	Prematurity	FLZ	Clinical improvement
Xia *et al.* ^17^	Wenzhou, China	30+5 w/M	1520	MEM	N	Y	PICC	Vaginal delivery	Prematurity, Intestinal necrosis	FLZ	Clinical improvement
		39+5w/F	2650	None	Y	Y	Umbilical venous catheter	Cesarean section	Dyspnea	FLZ	Clinical improvement
This study	Nanning, China	31+6w/M	1420	PEN, TZP, IPM	Y	Y	N	Cesarean section	Prematurity, Congenital laryngomalacia	FLZ	Clinical improvement

M = male; F = female; Y = yes; AMK = amikacin; AMP = ampicillin; VA = vancomycin; GM = gentamicin; CLO = cloxacillin; MEM = meropenem ; CPS = cefoperazone-sulbactam; IPM = imipenem; TZP = piperacillin-tazobactam; PEN = penicillin; NS = not specified; CVC = central venous catheters; PICC = peripherally inserted central catheter; FLZ = fluconazole; AMB = amphotericin B; MFG = micafungin; VRC = voriconazole; CAS = caspofungin.

As reported, the major risk factors of neonatal candidemia might involve premature patients, low birth weight, invasive therapeutic devices, the widespread use of broad-spectrum antimicrobial agents, and parenteral nutrition infusion^4,18^. Notably, our patient showed all these significant risk factors.

In our case, the patient showed a favorable clinical response to the fluconazole treatment despite the non-wild-type susceptibility of the isolate and its notably elevated MIC (>128 µg/mL), as in Khan ZU^3^. This highlights that *in vitro* AFST fails to always correlate with clinical outcomes, offering therapeutic challenges. This discrepancy may be attributed to unique pharmacokinetic and pharmacodynamic properties in neonates, warranting further investigation. Among 20 *C. haemulonii* species causing neonatal candidemia (including 19 cases in the literature and our case), 80% (16/20) of which had high MICs to amphotericin B (>2 µg/mL). According to a study of the China hospital invasive fungal surveillance net, 56% (36/64) of *C. haemulonii* isolates showed multidrug resistance^19^. A study from Escola Paulista de Medicina showed that all its *C. haemulonii* isolates that were collected in the previous 11 years showed high MICs for amphotericin B and fluconazole^2^. Similarly, all clinical 2002-2021 *C. haemulonii* complex isolates from France showed decreased *in vitro* susceptibility to amphotericin B and fluconazole^20^. The resistance of *C. haemulonii* represents a great challenge in the treatment of candidemia. Currently, no treatment guideline exists for invasive candidiasis by *C. haemulonii.* Fluconazole usually acts as a first-line antifungal agent for candidemia, especially in many low-income countries^20^. Amphotericin B was often used as salvage therapy^20^. However, cases in which neonates died from *C. haemulonii* candidemia despite treatment with fluconazole or amphotericin B have been reported^3,14^. The Infectious Diseases Society of America and the global guideline to diagnose and manage candidiasis recommend echinocandins as initial therapy for candidemia^21,22^. Given the low MICs observed for echinocandins, these agents may represent the optimal first-line treatment for candidemia by *C. haemulonii* prior to AFST results. This recommendation agrees with clinical evidence on the successful outcomes in neonatal cases treated with echinocandins^15^. Taken together, further studies evaluating antifungal agents for the management of candidemia are essential.

*C. haemulonii* was a pathogen associated with outbreaks of nosocomial candidemia in premature newborns^3,14^, as might be associated with its skin carriage and colonizing foreign devices^20^. Studies have reported that *C. haemulonii* and *C. auris* shared some pathogenicity-related traits, such as adhesion on prosthetic materials, multidrug resistance, and phenotypic switching^20^.

## CONCLUSION

In conclusion, we reported a case of neonatal candidemia by *C. haemulonii* that was successfully treated with fluconazole despite its *in vitro* resistance given its similarity to other species of the same complex and *C. auris* in morphology. This study recommends matrix-assisted laser desorption/ionization time-of-flight mass spectrometry or molecular methods to identify species. Correct AFST will improve clinical prognoses. Echinocandins might configure the drug of first choice to treat it. Pediatricians in neonatal intensive care unit should remain alert to candidemia in premature newborns with low birth weight and enhance experiential administration control and the management of invasive procedures.
